# Validation of the South Korean Version of the Beliefs about Emotions Scale

**DOI:** 10.1186/s40359-021-00713-4

**Published:** 2022-01-05

**Authors:** Gahyun Park, Jeong Han Kim, Dong Hun Lee

**Affiliations:** 1 Traumatic Stress Center, Department of Education, Sungkyunkwan University, 51112 Hoam Hall, 25-2, Sungkyunkwan-ro, Jongno-gu, Seoul, 03063 South Korea; 2 School of Rehabilitation Services and Counseling, The University of Texas Rio Grande Valley, Edinburg, Texas USA

**Keywords:** Beliefs, Emotions, Validation, South Korea

## Abstract

**Background:**

Beliefs about the unacceptability of experiencing or expressing negative emotions can contribute to diverse psychological symptoms and associated with poor treatment outcomes and low treatment attempts. The Beliefs about Emotions Scale (BES) was developed to assess such beliefs based on the cognitive-behavioral models; however, no study has reported on the psychometric properties of the BES in Korea. The present study aimed to cross-culturally adapt and validate the BES for the Korean population (BES-K).

**Methods:**

The BES-K was administered to 592 Korean adults (323 men and 269 women) aged 20–59 years. Exploratory and confirmatory factor analysis were used to assess the factor model of the scale. Pearson correlation coefficients were used to evaluate the relationships between the BES-K and other psychological measures.

**Results:**

The result showed a two-factor model of the BES-K, with Factor 1 relating to Interpersonal and Factor 2 representing Intrapersonal aspects. The scale had significant yet moderately low correlations with measures of depression, anxiety, and difficulties in emotion regulation.

**Conclusion:**

The BES-K is a useful instrument in evaluating the beliefs about emotions in the Korean population.

## Background

Processing emotional states has a crucial role in a variety of health conditions. There is growing evidence that beliefs about the unacceptability of experiencing and expressing negative emotions are related to diverse clinical problems and symptoms such as depression [1], anxiety [2], somatic symptoms [3], eating disorders [4], chronic fatigue syndrome [5], and irritable bowel syndrome [6]. Cognitive behavioral models propose that such beliefs contribute to the etiology and maintenance of various symptoms and disorders [7–9]. In addition, it has been argued that these beliefs are a transdiagnostic vulnerability factor that gives rise to various problems and is associated with poor treatment outcomes and low treatment attempts [10, 11].

Cognitive behavioral models of chronic fatigue syndrome suggest that these beliefs may develop in some venerable individuals nurtured in an environment where the display of negative emotions and difficulties was met with punishment or lack of positive feedback [4, 8, 10]. Believing in positive attitude such as “being happy” and “never giving up” is overvalued, while displaying any negative thoughts and emotions is not tolerated [4]. This emotionally invalidating environment leads to the beliefs that experiencing and expressing negative thoughts or emotions are unhelpful and unacceptable, cause adverse social consequences, and thus should be avoided, suppressed, or at least not overtly revealed [4].

These beliefs contribute to difficulties in many ways [10]. People who have such beliefs have difficulty in properly understanding and taking care of themselves, as there is substantial evidence that emotions help people to reorganize, understand, and interpret their subjective state [12]. Moreover, while experiencing distress, these individuals may engage in self-silencing behaviors to create or maintain safe and close relationships by “putting on a brave face”. Silencing certain negative thoughts and emotions and not seeking help have a counterproductive effect of blocking social support that may otherwise be beneficial [4, 10, 13, 14]. Additionally, suppression or avoidance of unwanted thoughts and emotions ironically increases them, which in turn results in higher distress and associated mental and physical symptoms such as numbness, fatigue, and bowel disturbance [10].

Few studies have attempted to measure beliefs about emotions. There are several scales measuring behavioral responses that are closely related to beliefs about emotions, such as avoidance and suppression [15], but few studies have evaluated the underlying beliefs that lead to those behaviors [10]. It is true that some emotion regulation questionnaires that measure individuals’ strategies to modulate negative emotions partly contain items evaluating beliefs about emotions [16, 17]. However, as most of the items assess behavioral strategies in time of experiencing negative emotions, such as “engaging in goal-directed behaviors” and “refraining from impulsive behaviors”, these scales have rarely been used to measure beliefs about the emotions themselves. Therefore, a new questionnaire that can briefly assess such beliefs has been required.

Rimes and Chalder [10] developed the Beliefs about Emotions Scale (BES) to assess negative beliefs about experiencing and expressing negative thoughts and emotions based on cognitive behavioral models in the United Kingdom. The BES items were designed and selected to illustrate the types of such beliefs identified in clinical reports and cognitive behavioral models [10]. The BES is a 12-item scale, preferably brief and easy to use. It is unidimensional with adequate internal consistency (Cronbach’s α = 0.91) and validity in its development study [10]. The BES was cross-culturally validated in another cultural context—Brazil, and the Brazilian Portuguese version [11] showed a two-factor model with fair internal consistency (Cronbach’s α = 0.86) and validity. The first factor (Item 1, 2, 3, 5, 6, 8, 9, 10, 11) accounted for “emotions and their expressions as signs of weakness”, and the second factor (Item 4, 7, 12) was linked to “emotional self-control”. The disagreement in the factor structures of the two versions may reflect cultural differences [11], which lays the groundwork for the discussion on cultural differences in beliefs about emotions.

The BES has been utilized in various studies exploring and intervening in individuals with chronic fatigue syndrome [5, 18–20], irritable bowel syndrome [6, 14, 21], anorexia and bulimia nervosa [4], perfectionism [22–24], depression [20, 23, 24], anxiety [20], and fibromyalgia [21, 25].

According to Rimes and Chalder [10], the benefit of measuring these beliefs that derive behavioral responses (e.g., suppression and avoidance), rather than evaluating behaviors themselves, is that individuals can easily assess and self-report their beliefs. For instance, individuals may not recognize the extent of their suppression or avoidance behaviors if these behaviors are overlearned and become relatively automatic behavioral responses. Another advantage is that measuring beliefs is more clinically useful as different forms of modern cognitive psychotherapy and psychoeducation try to address and modify these negative beliefs about emotions and increase psychological flexibility [10]. For example, third wave cognitive behavioral approaches based on mindfulness such as MBSR (Mindfulness Based Stress [26, 27]), DBT (Dialectical Behavior therapy [28]), ACT (Acceptance and Commitment Therapy [29]), and MBCT (Mindfulness Based Cognitive Therapy [30]) develop nonjudgmental awareness and radical acceptance of emotions, thoughts, and bodily sensations.

To date, no studies have reported on the psychometric properties of the BES in Korea. This study aimed to cross-culturally adapt and validate the BES in a Korean population sample (BES-K). In Korea, without a reliable and valid scale to measure beliefs about emotions, relevant studies have been quite limited. For instance, given that modern cognitive behavioral models state such beliefs are key variables in the etiology and maintenance of a variety of symptoms and disorders including chronic fatigue syndrome, irritable bowel syndrome, and eating disorders [7–9], it is surprising that no study exploring such beliefs in individuals with these symptoms can be identified in the existing Korean literature. Thus, there has been a need for a proper scale to evaluate beliefs about emotions that can encourage relevant studies and interventions. Further, to the best of our knowledge, there is only one study analyzing the factorial structure of the BES for other cultural contexts, the Brazilian Portuguese version [11]. This study also aimed to contribute to the discussion on the cultural differences of beliefs about emotions by providing additional cultural data on such beliefs.

Therefore, the objectives of this study were: (1) to investigate the psychometric properties of the BES-K using both exploratory and confirmatory factor analyses (EFA and CFA). Performing EFA was necessary instead of doing CFA directly, considering that the factor structures of the previous two versions (the original English and the Brazilian version) are not congruent, and people in different cultures differ in their emotional experiences and expressions [31–33]; (2) to compare factor structure models found in the previous studies [10, 11] with the BES-K model; and (3) to examine the convergent validity of the BES-K by examining associations with other criterion scales.

## Methods

### Participants and sampling

As a part of a two-year government funded research project, the national online survey was implemented according to the Korean population census standard, considering sociodemographic factors such as sex, age, and residential area. The inclusion criteria for the sample were: 1) age between 20 and 59 years and 2) reading and writing proficiency in Korean. Originally, 845 participants enrolled in the survey; 608 participants completed the survey (72.0%). The exclusion criterion was insufficient effort responding (e.g., “leaving items unanswered” and “using the same response repeatedly”) [34]. The inclusion of these responses into the dataset can have various unexpected and unwanted effects on relationships being examined; thus, the removal of such responses is suggested [35, 36]. As our Internet-based survey was designed such that the participants could not skip any questions without ticking their responses, there were no participants who left items unanswered. However, 16 participants ticked the same response (number) consecutively and were therefore excluded. The final sample consisted of 592 participants (54.6% men and 45.4% women).

This study received ethical approval from the Institutional Review Board (IRB) to which the researchers are affiliated. All study protocols were performed in accordance with the Declaration of Helsinki. A survey was conducted between July and August 2019 by a confidential Internet-based survey company that utilizes a firewall (WAF) and secure socket layer (SSL) on its securities and encryption. The survey took approximately 30 min to complete, and vouchers worth two dollars were provided to the participants as compensation.

### Instruments

#### The Beliefs about Emotions Scale (BES)

The original BES [10] has 12 items that assess beliefs about the unacceptability of experiencing and expressing negative thoughts and emotions (e.g., “I should be able to control my emotions”). Items are rated on a 7-point Likert scale from 0 to 6, with high scores demonstrating more unacceptability of negative thoughts and emotions. The original BES has a single factor with good internal consistency (Cronbach’s α = 0.91).

To develop the Korean version of the BES (BES-K), the following steps were taken based on the cross-cultural adaptation process guidelines: (a) translation, (b) back-translation, (c) committee review, (d) pretesting, and (e) final consensus [37, 38]. (a) After obtaining permission to validate the BES from the original authors, two Korean researchers fluent in both languages translated the original English BES into Korean. (b) A bilingual researcher familiar with both cultures and languages performed back-translations. (c) The research team comprising 20 trained counselors with master’s and Ph.D. degrees in counseling psychology compared it to the original English version to discuss whether there were any discrepancies. There was a semantic discrepancy on idiom “think less of me” in Item 3. Modification was made to this item until there was no feedback from researchers. Further, some researchers suggested a more natural Korean word for “a sign of weakness” in Item 6 and Item 11. The research team came to a consensus on the suggested word, so modifications were made to the items. (d) A pilot test was conducted on a sample of 30 undergraduate and graduate students to ensure readability and comprehensibility of the scale. After completing the questionnaire, they were also asked about their thoughts or responses to the items. Based on these comments, minor revisions were made to improve the sentence fluency of the items until the research team reached a consensus. (e) Finally, a professor at an American university (Ph.D. in rehabilitation psychology) and one at a Korean university (Ph.D. in counseling psychology) confirmed the final version tested in the present study.

#### The Brief Symptom Inventory-18 (BSI-18)

The BSI-18 [39] has 18 items that assess psychological distress during the past seven days. It includes items assessing depression (e.g., “Feeling no interest in things”), anxiety (e.g., “Feeling tense or keyed up”), and somatization (e.g., “Nausea or upset stomach”). Each item is rated on a 5-point Likert scale from 0 to 4, and all three dimensions have six items each. Possible total scores vary from 0 to 72 points, with high scores indicating greater psychological distress. The BSI-18 showed fair internal consistency (Cronbach’s α = 0.84, 0.79, and 0.74 for depression, anxiety, and somatization, respectively). The Korean version of the BSI-18 [40] also reported fair internal consistency (Cronbach’s α = 0.80, 0.81, and 0.73 for depression, anxiety, and somatization, respectively). Based on the significant positive relationship of the BES with depression and anxiety from previous studies [10, 11], only these two subscales were used in the present study. Cronbach’s α of the BSI-18 in this study was 0.90 for depression and 0.91 for anxiety.

#### The Difficulties in Emotion Regulation Scale-16 (DERS-16)

The DERS-16 [41] has 16 items that assess an individual’s typical level of emotion regulation. It assesses five dimensions of emotion regulation difficulties: non-acceptance of negative emotions (e.g., “When I am upset, I feel like I am weak”), inability to engage in goal-directed behaviors when distressed (e.g., “ When I am upset, I have difficulty thinking about anything else”), difficulties controlling impulsive behaviors when distressed (e.g., “When I am upset, I become out of control”), limited access to emotion regulation strategies perceived as effective (e.g., “When I am upset, I start to feel very bad about myself”), and lack of emotional clarity (e.g., “I am confused about how I feel”). Items are rated on a 5-point Likert scale from 1 to 5, and possible total scores vary from 16 to 80, with high scores reflecting more difficulty in emotion regulation. The DERS-16 showed good internal consistency (Cronbach’s α = 0.92). The psychometric properties of the Korean version of the DERS-16 [42] were as strong as the original (Cronbach’s α = 0.92). Cronbach’s α of the DERS-16 in this study was 0.93.

### Statistical analysis

Data analyses in this study were performed using the SPSS version 21.0 and AMOS version 21.0. First, the distribution, central tendency, and dispersion of all variables were inspected. Kline [43] recommended that none of variables should exceed the standard value of skewness (≤|2.0|) and kurtosis (≤|4.0|) to verify a fair level of normality of the data. Second, a split-half method [44, 45] was chosen to allow for independent EFA and CFA. The dataset was randomly split into two halves. One half (subsample I) was a training sample where EFA would be performed to discover the number and nature of latent factors inherent to the BES, and the other half (subsample II) was a testing sample where the structural model identified from EFA would be tested through CFA. Third, EFA using maximum likelihood estimation (MLE) was performed on the 12 BES items in subsample I (n = 270). Oblique rotation of the items was done because it has advantages if one or more components are somehow related rather than independent [46]. Scree test [47] and eigenvalue criterion [48] were employed to determine the appropriate number of factors to retain. Items with a factor loading above 0.40 were retained. Fourth, CFA was performed on the retained BES items in subsample II (n = 322) and other alternative models proposed in previous studies. Model fit was evaluated based on chi-square (χ^2^/df), comparative fit index (CFI), Tucker Lewis index (TLI), normed fit index (NFI), standardized root mean square residual (SRMR), and root mean square error of approximation (RMSEA), following the standards of previous studies [46, 49–51]. Akaike information criterion (AIC) was also employed to compare several non-nested models. Finally, we examined the validity of the scale. The convergent validity of the scale was evaluated by Pearson correlation coefficients.

## Results

### Socio-demographic characteristics

The participants were 592 Korean adults (323 men and 269 women) aged between 20 and 59 years (*M* = 39.7, *SD* = 9.7). The age distribution was as follows: 20.6% were 20–29 years, 27.7% were 30–39 years, 31.3% were 40–49 years, and 20.4% were 50–59 years. Most of the participants (87.0%) had received or had been receiving post-secondary education (e.g., college or university education), with only 12.5% having received secondary education. Moreover, most of the participants (79.4%) were employed or self-employed, 7.3% were housewives, 5.9% were unemployed, and 5.6% were students. Subsample I consisted of 270 participants (55.2% men and 44.8% women) with a mean age of 40.6 years (*SD* = 9.8), and Subsample II consisted of 322 participants (54.0% men and 46.0% women) with a mean age of 39 years (*SD* = 9.6).

### Preliminary analysis

All variables were first examined on normal distribution. None of them exceeded the standard value of skewness (≤|2.0|) and kurtosis (≤|4.0|).

### Exploratory factor analysis

The suitability of the data for factor analysis was inspected by assessing the sample fits of the Kaiser–Meyer–Olkin and the Bartlett’s sphericity test. The Kaiser–Meyer–Olkin sample fit was 0.89, exceeding the acceptable value of 0.6, and Bartlett’s sphericity test reached statistical significance (χ^2^ = 1,497.097, df = 66, p = 0.00). The results indicated the factorability of the correlation matrix. The 12 items of the BES were subjected to maximum likelihood estimation (MLE) and Oblimin rotation. The following criteria were used to determine the most appropriate number of factors: (a) a minimum eigenvalue of 1.0 criterion, (b) a minimum of four items in each factor, (c) deletion of items with factor loadings less than 0.40, and (d) conceptual coherence of each factor [52]. As shown in Table 1, a two-factor structure was suggested, with a total explained variance of 57.6%. Factor loadings of Item 1 were less than 0.40 on both factors, and it was therefore deleted. This resulted in seven items in the first subscale and four items in the second subscale. However, the two-factor model of the BES-K was not consistent with the dimensionality of the original English version. Factor 1 of the BES-K explained the beliefs about emotions at the interpersonal and social level, emphasizing that individuals often regulate and control emotions considering the negative social consequences of their emotions; Factor 2 explained the beliefs at the intrapersonal and personal level, focusing on whether people should be able to control or cope with negative thoughts and emotions. Therefore, Factor 1 was labeled as Interpersonal, and Factor 2 was labeled as Intrapersonal.

**Table 1:** The two-factor structure from exploratory factor analysis (EFA) in sample I (n = 270)

The BES items	Factor 1	Factor 2
Eigenvalue	5.68	1.22
Percentage of variance explained	47.37	10.18
6. If I show signs of weakness then others will reject me	**.934**	− .176
2. If I have difficulties I should not admit them to others	**.755**	− .104
11. It would be a sign of weakness to show my emotions in public	**.721**	.086
9. To be acceptable to others, I must keep any difficulties or negative feelings to myself	**.689**	.108
3. If I lose control of my emotions in front of others, they will think less of me	**.650**	.156
5. If I am having difficulties it is important to put on a brave face	**.549**	.157
12. Others expect me to always be in control of my emotions	**.446**	.275
1. It is a sign of weakness if I have miserable thoughts	.398	.252
7. I should not let myself give in to negative feelings	.107	**.678**
8. I should be able to cope with difficulties on my own without turning to others for support	.189	**.616**
4. I should be able to control my emotions	− .092	**.610**
10. It is stupid to have miserable thoughts	.191	**.465**

Factor loadings equal to or greather than .40 are in boldface

A cut-off score of item loading was .40

### Confirmatory factor analysis

**Fig. 1:**
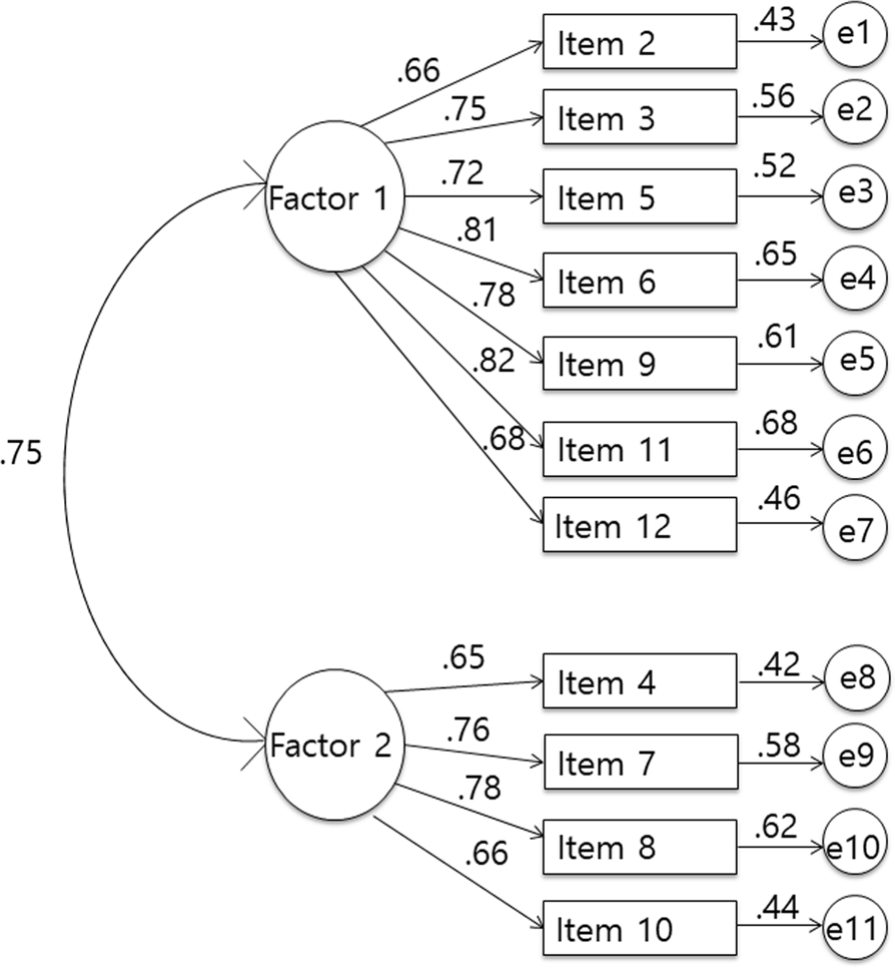
Confirmatory factor analysis of the BES-K

Confirmatory factor analysis was performed on the BES-K model (11 items, with two factors, e.g., Interpersonal and Intrapersonal), as well as other alternative models. Alternative models were included to check whether they might better represent our data. Table 2 shows that only the BES-K model had acceptable model fit indices. For the comparison of the four models, we used AIC value, with lower value representing a better fit. The lowest AIC value was observed in the BES-K model. Further, Fig. 1 shows that the standardized regression weights of all 11 items of the BES-K were between 0.65 and 0.82, and each of them reached statistical significance (*p* < 0.001).

**Table 2:** Confirmatory factor analysis for four forms of the BES

Variable	χ^2^	df	CFI	TLI	NFI	RMSEA	SRMR	AIC
Model 1	174.554	43	.928	.907	.907	.098 [.083–.113]	.051	220.554
Model 2	299.846	44	.859	.824	.840	.135 [.120–.149]	.071	343.846
Model 3	364.775	54	.843	.808	.822	.134 [.121–.147]	.072	412.775
Model 4	342.495	53	.854	.818	.833	.130 [.117–.144]	.070	392.495

Model 1 (Korean model, 11 items, two factors); Model 2 (11 items, one factor); Model 3 (original English model, 12 items, one factor); Model 4 (Brazilian model, 12 items, two factors); χ^2^/df, chi-square/degrees of freedom; CFI, comparative fit index; TLI, Tucker-Lewis index; NFI, normed fit index; RMSEA, root mean square error of approximation; SRMR, standardized root mean square residual; AIC, Akaike information criterion

### Internal consistency

Internal consistency of the BES-K was calculated for the scale as a whole (Cronbach’s α = 0.90) as well as for each subscale (Cronbach’s α = 0.89 for Interpersonal and 0.78 for Intrapersonal), indicating fair reliability.

### Validity

Convergent validity was evaluated by analyzing Pearson correlation coefficients between the BES-K and other questionnaires, namely, the BSI-18, and the DERS-16. The BES-K showed significant yet moderately low correlations with the BSI depression (r = 0.27, p < 0.01), the BSI anxiety (r = 0.21, p < 0.01), and the DERS-16 (r = 0.36, p < 0.01). Interpersonal subscale also showed significant positive correlations with the BSI depression, (r = 0.33, p < 0.01), the BSI anxiety, (r = 0.29, p < 0.01), and the DERS-16 (r = 0.47, p < 0.01), whereas Intrapersonal subscale was not significantly related to them.

## Discussion

The BES, a brief measure of beliefs about experiencing and expressing negative thoughts and emotions, was developed in the United Kingdom and widely used in various studies, including chronic fatigue syndrome, irritable bowel syndrome, anorexia nervosa, bulimia nervosa, perfectionism, depression, anxiety, and fibromyalgia. The BES was cross-culturally adapted to another culture, Brazil, and the factor structure of this Brazilian Portuguese version was found to be different from the original English version, laying the groundwork for the discussion on cultural differences in beliefs about emotions. The present study aimed to contribute to the discussion on cultural differences in beliefs about emotions by evaluating the scale in the Korean context.

Running both EFA and CFA was necessary because they would identify and verify the nature of latent factors of the scale in the Korean context. Our result demonstrates that the BES-K is divided into two factors: Interpersonal, consisting of seven items (Item 2, 3, 5, 6, 9, 11, 12) and Intrapersonal, consisting of four items (Item 4, 7, 8, 10). The Interpersonal factor evaluates the beliefs about emotions at the social and interpersonal level, focusing on the negative social consequences of emotions (e.g., “If I lose control of my emotions in front of others, they will think less of me”, “To be acceptable to others, I must keep any difficulties or negative feelings to myself”); and Intrapersonal factor measures the beliefs at the personal and intrapersonal level, focusing on whether people should be able to control or cope with negative thoughts and emotions (e.g., “I should be able to control my emotions”, “I should not let myself give in to negative feelings”).

Previous studies reported that emotional experiences and expressions fundamentally differ among cultures [31–33]. In collectivist cultures, emotions are regarded as relational phenomena reflecting the state of relationships rather than a unique personal inner state; thus, emotional expression is adjusted and sometimes suppressed according to the social context and self-other relationships [53–55]. For example, there are two Korean words, “*Nun-chi* (meaning being conscious to others)” and “*Che-myon* (meaning having a social face within a group)”, which describe adjusting personal emotions and behaviors in accordance with social context and relationship. Within the Korean society, one is considered socially inept and awkward when one is described to lack “*Nun-chi*” or “*Che-myon*”. Koreans are educated to control their emotions based on social cues and others’ expectations and are highly concerned about potential negative consequences of expressing their emotions. These relationship-oriented characteristics of Koreans might explain why the BES-K is divided into two different constructs: Interpersonal and Intrapersonal.

In comparison to the original English and its Brazilian adaptation, all three versions have different factor structures. Unlike the unidimensionality of the original English version, a two-factor model was shown in the Brazilian Portuguese version, where the first factor (Item 1, 2, 3, 5, 6, 8, 9, 10, 11; considering emotions and their expressions as signs of weakness) is distinctive from the second factor (Item 4, 7, 12; emotional self-control). The authors explained that emotional control can be a different aspect of emotional expression in a Latin American culture, which favors emotional expression and is more expressive than European culture [11]. In the Korean version, another two-factor model was confirmed. Two versions differ in Item 1, 8, 10, and 12. Further, the interpretation of each factor in the two versions was somewhat different. In the Korean version, the cause of regulating, controlling, or coping with emotions (e.g., whether it is an interpersonal cause or intrapersonal cause) was an important criterion between the two factors. However, in the Brazilian version, although the interpretation was not explained in detail, we can assume emotional self-control was a critical criterion between the two factors. For instance, in Item 12 (“Others expect me to always be in control of my emotions”), Korean focused on “others expect”, whereas Brazilian focused on “control”.

The relationship between the BES-K and the criterion scales was largely in line with previous studies. The correlations for the Korean sample were significant yet lower than the original BES and similar to the Brazilian version, particularly in terms of depression and anxiety. All three versions showed small-to-medium correlations with the criterion scales. Some researchers explained that these significant yet not strong correlations between the BES and other psychological symptoms imply the idea that negative beliefs about emotions may represent a transdiagnostic vulnerability factor contributing to diverse clinical problems rather than having a specific association with a certain problem [10, 11]. When examining the two subscales of the BES-K, Intrapersonal subscale was not significantly related to the criterion scales.

To understand this discrepancy, the research team to which authors are affiliated re-checked the three versions of the BES. We found that some researchers interpreted Item 4 (“I should be able to control my emotions”) and Item 7 (“I should not let myself give in to negative feelings”) as their adaptive ability to manage and regulate negative emotions not only in the Korean version but also in the original English version. This led us to suggest a possible assumption that these items might be open to interpretation among cultures due to cultural differences in emotional processing. As mentioned above, in Korea whose values and mentalities still heavily rely on the ideas of Confucianism such as harmony, people are socialized to control personal emotions and behaviors in accordance with social context and others. People are considered as “mature” if they maintain social harmony and good relationship by controlling and adjusting their emotions. Thus, in Korea, where emotional control is highly valued and recommended, Item 4 and 7 might be understood as desirable and adaptive ability to control and manage emotions, rather than maladaptive emotional suppression or avoidance, thereby diminishing the correlations with the criterion scales. This discrepancy was also found in Factor 2 (emotional self-control; Item 4, 7, 12) in the Brazilian version, and the authors of the Brazilian version also explained that this might be related to cultural and contextual differences [11]. Therefore, we assumed the fact that the Intrapersonal factor did not correlate with anxiety and depression was related to the cultural differences in emotional processing, but this issue needs to be further investigated in future studies.

Some limitations of the present study should be addressed. First, participants in the present study were recruited online and the age range of the participants was limited to 20–59 years. Therefore, it is hard to generalize and apply the results to different age groups and clinical populations. Further studies are needed to validate the BES-K in different age groups (e.g., adolescents, university students, and senior citizens) and diverse clinical sample (e.g., individuals with chronic fatigue syndrome, and irritable bowel syndromes, and eating disorders). Second, causal inferences between the BES-K and other psychological variables could not be drawn given the cross-sectional design of the study. Experimental or longitudinal designs are required to understand the cause-and-effect relationships between variables.

This study was the first to our knowledge to adapt and psychometrically evaluate beliefs about emotions in Korea. As such beliefs represent a transdiagnostic vulnerability factor contributing to diverse clinical problems [10], the BES-K would allow better understanding of various symptoms and encourage relevant studies in Korea. Furthermore, in therapeutic settings, as various current cognitive behavior psychotherapies focus on addressing and making interventions for such beliefs, the BES-K would be practically useful in addressing the beliefs that drive maladaptive behaviors, helping developing alternative behavior responses, and evaluating the effectiveness of therapeutic interventions. In addition, as emotions are understood and experienced in a fashion analogous to the dominating ideas and values of each culture wherein emotions occur [33], the BES-K would contribute to further discussion on cultural differences in beliefs about emotions, as well as emotional processing.

## Data Availability

The data used in the present study are available from the corresponding author upon reasonable request.

## Abbreviations

BES: Beliefs about Emotions Scale
BES-K: Korean version of the Beliefs about the Emotions Scale
EFA: Exploratory factor analysis
CFA: Confirmative factor analysis
DERS-16: Difficulties in Emotion Regulation Scale-16
BSI-18: The Brief Symptom Inventory-18
MLE: Maximum likelihood estimation
CFI: Comparative fit index
TLI: Tucker–Lewis index
NFI: Normed fit index
SRMR: Standardized root mean square residual
RMSEA: Root mean square error of approximation
AIC: Akaike information criterion
